# Fading red? No evidence that color of trunks influences outcomes in the ultimate fighting championship (UFC)

**DOI:** 10.3389/fpsyg.2013.00643

**Published:** 2013-09-19

**Authors:** Thomas V. Pollet, Leonard S. Peperkoorn

**Affiliations:** Department of Social and Organizational Psychology, VU University AmsterdamAmsterdam, Netherlands

**Keywords:** red, human performance, contests, ultimate fighting championship, color

In fish, reptiles, birds, and primates it has been documented that the color red plays an important role in dominance and threat displays, with the more intimidating individual being associated with winning contests (Pryke, 2009 for review). A much debated paper by Hill and Barton (2005) sparked off research concerning the influence of red attire on combat sports outcomes in humans, arguing that red could also play a role in these dominance contests. Hill and Barton (2005) presented evidence from four combat sports during the 2004 Olympics showing significantly higher win-ratios for red as opposed to blue outfits, suggesting that red clothing could heighten the likelihood of winning. The findings were explained as psychological effects on either one or both of the contestants originating from evolutionary and cultural associations between red and dominance as well as between red and aggression. A study by Feltman and Elliot (2011), using a scenario methodology, found preliminary evidence for both wearing and viewing effects of red equipment on perceptions of dominance and threat.

However, a replication attempt of Hill and Barton (2005) relying on the 2008 Olympics did not support a performance enhancing effect of red (Seife, n.d.). Moreover, the statistical assumptions for tests in Hill and Barton (2005) could have been violated (non-independence) and selection bias in allocation of red and blue is possible (Seife, n.d.). In addition, Rowe et al. (2005) proposed an alternative explanation for Hill and Barton's (2005) findings. After finding a similar winning bias for *blue* over white in judo competitions Rowe et al. (2005) argued that visibility differences between contestants may be responsible for observed effects since red was not represented in these judo fights. Dijkstra and Preenen (2008) indicated that the Rowe et al. (2005) study was confounded and found no winning blue effect after controlling for potential biases. The “winning red” effect in combat sports was supported, however, by Hagemann et al. (2008), who argued that red can lead to a perceptual bias in referees. As predicted, referees were found to award significantly more points to taekwondo competitors in red rather than blue apparel.

The present study aims to conceptually replicate the effects found by Hill and Barton (2005) by testing for a “winning red” effect based on the color of shorts (trunks) in the ultimate fighting championship (UFC) (www.ufc.com), a televised mixed martial arts competition. The UFC has very limited rules for combat and hence provides an opportunity to test whether the “winning red” effect generalizes to a less regulated combat sport. Finally, the effect of color on referees' decisions and clear fight outcomes will be examined.

For episodes 118–148 (210 matches) we coded the base color of the shorts worn by the fighters (for description of sample: Pollet et al., in press). Sometimes shorts have multiple colors, a primary and a secondary, but we coded the “base” color, typically also displayed at the bottom of the screen. The predominant colors were Black, White, Red, Blue, and “Other,” which included rarely chosen colors: Brown (*n* = 4), Camouflage (*n* = 3), Gold (*n* = 1), Gray (*n* = 5), Green (*n* = 6), Pink (*n* = 2), Purple (*n* = 3), and Yellow (*n* = 2). The UFC does not have colored gloves as in boxing but the black gloves do have either a small blue or red band. These were not coded, as the bands on the predominantly black gloves are substantially smaller than the trunks. Three draws were excluded from our analyses. Analyses were conducted in SPSS 20.0 and were two-tailed. Sensitivity power analysis via G*Power 3.1 (Faul et al., 2007) indicates sufficient power to detect a relatively weak effect (*g* = 0.18) in our sample (Binomial test, two-tailed, α = 0.05; Power = 0.8).

**Figure 1:**
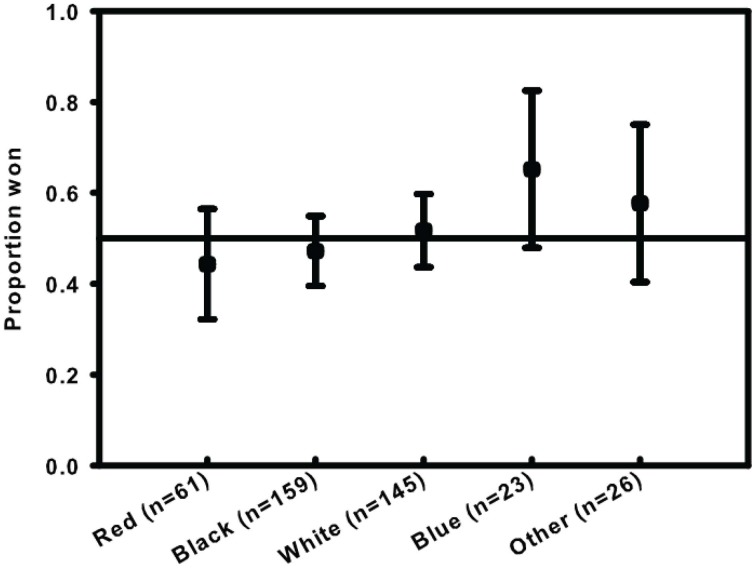
**Proportion of UFC fights won by color of shorts.** Ninety-five percent confidence intervals are based on Agresti–Coull method. Reference line is 0.5.

A simple binomial test did not support a winning red effect (27 wins out of 61; Binomial test *p* = 0.443; Bayes factor = 4.245 in favor of 0.5 win rate; Rouder, n.d.). In a Generalized linear model (GZLM) with binomial link, color (5 categories) did not account for fight outcome (Win/Loss; Wald χ^2^ = 4.153, *p* = 0.386; Figure 1).

Incidentally, we also tested a multilevel logit model taking into account a nested structure based on “fighter,” as the same fighter can appear multiple times and could have a preference for shorts. In this model, color also did not account for fight outcome, *p* = 0.387. Similarly, conducting a GZLM separately for jury decisions and “clear” outcomes (Technical Knockout, Knockout, submission or tap-out) did not affect the effect of color on fight outcome (*p* = 0.941 and *p* = 0.472, respectively).

In summary, there is no support for a “winning red” effect. There are several limitations to the current study. For example the trunks might be simply less salient than headgear or the sports attire studied by Hill and Barton (2005). Moreover, these trunks have sponsoring, usually in bright neon fonts, which could make red less salient. There is no reason to assume, however, that red shorts would attract more sponsoring than, the more common black or white shorts. While the base color is clearly salient, the logos of sponsors could, however, dilute the “winning red” effect. The trunks we coded also have secondary colors, which could interfere with the base color. We believe this is unlikely however, to explain away our findings. For “red,” 41 out of 61 shorts had red as their only color (apart from sponsoring) and of these 41, only 19 won their fight (Binomial test; *p* = 0.755; Bayes Factor = 4.673). It therefore seems that considering secondary colors will not alter our key finding. For now we have also assumed that fighters (or their managers) choose their shorts but currently no data are available on who picks their shorts. However, there is nothing to suggest systematic bias in the UFC with regards to assigning shorts. Therefore, we conclude that currently there is no evidence of a “winning red” effect in the UFC. While there is a clear-cut evolutionary rationale as to why red should matter for dominance contests in certain species, more data are needed on whether and when red matters in combat sports.
